# Intraosseous Lipomatous Meningioma

**DOI:** 10.1155/2015/482140

**Published:** 2015-01-26

**Authors:** Lauren Kim, Christopher Huang, Adrienne L. Morey, Mark J. Winder

**Affiliations:** ^1^Department of Neurosurgery, St Vincent's Hospital, 390 Victoria Street, Darlinghurst, NSW 2010, Australia; ^2^Department of Anatomical Pathology, St Vincent's Hospital, 390 Victoria Street, Darlinghurst, NSW 2010, Australia; ^3^Department of Neurosurgery, St Vincent's Clinic, 438 Victoria Street, Darlinghurst, NSW 2010, Australia

## Abstract

A 49-year-old man with intermittent headaches and right sided parietal lump was found to have an intraosseous right parietal lesion on computed tomography (CT) and magnetic resonance imaging (MRI). A stereotactic craniectomy and excision of the lesion were performed with histopathology confirming features consistent with primary lipomatous meningioma with intraosseous extension. Lipomatous meningiomas are very uncommon subtype of meningiomas, with ongoing discussions as to their true pathogenesis. To our knowledge this case represents the first reported case of a lipomatous meningioma with predominant intraosseous extension.

## 1. Case Report

A 49-year-old Caucasian male who works as a joiner presented with intermittent headaches and a progressively increasing right sided parietal lump. He denied any history of trauma or infective process and past medical history was unremarkable. The physical examination was normal apart from a palpable nonfluctuating, nontender mass overlying the right parietal region.

**Figure 1:**
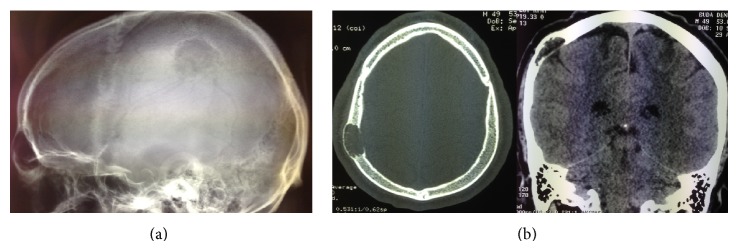
(a) Skull X-ray showing bony erosion. (b) CT brain showing inner table erosion and thinning of the outer table from the intraosseous lesion.

**Figure 2:**
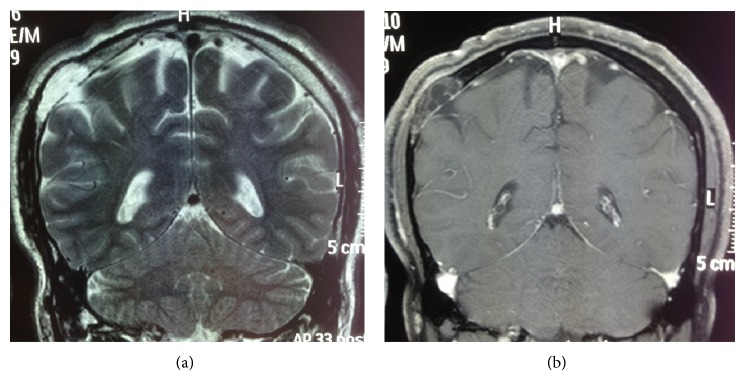
(a) T2 MRI showing high signal within the intraosseous lipomatous meningioma and dural defect. (b) T1 MRI with gadolinium observing heterogenous uptake within the lipomatous meningioma with no obvious intraparenchymal extension.

Skull X-ray, CT brain, and MRI brain revealed a single solitary lytic lesion within the right parietal bone with immense thinning of the outer table and erosion through the inner table without intracranial extension (Figure 1), although a small discontinuity in the dura mater was evident on MRI. The lesion demonstrated high signal on T2 weighted imaging (T2WI) and mixed low and normal signal intensity on T1 weighted sequences (Figure 2). There was no diffusion restriction and heterogeneous contrast enhancement with gadolinium was seen within the lesion. A bone scan demonstrated active uptake through the lesion.

The patient underwent a stereotactic craniectomy of the right parietal lesion. A wide craniotomy was performed and upon elevation of the bone flap a small defect in the dura was present with what appeared to be an arachnoid granulation, consistent with the observations on the MRI. A pale white, well-delineated tumour could be seen eroding through the bone. A dural resection was performed around a small attached tan coloured tumour and the brain was inspected with no evidence of tumour involvement. Ensuring clear margins, duraplasty and cranioplasty were performed.

**Figure 3:**
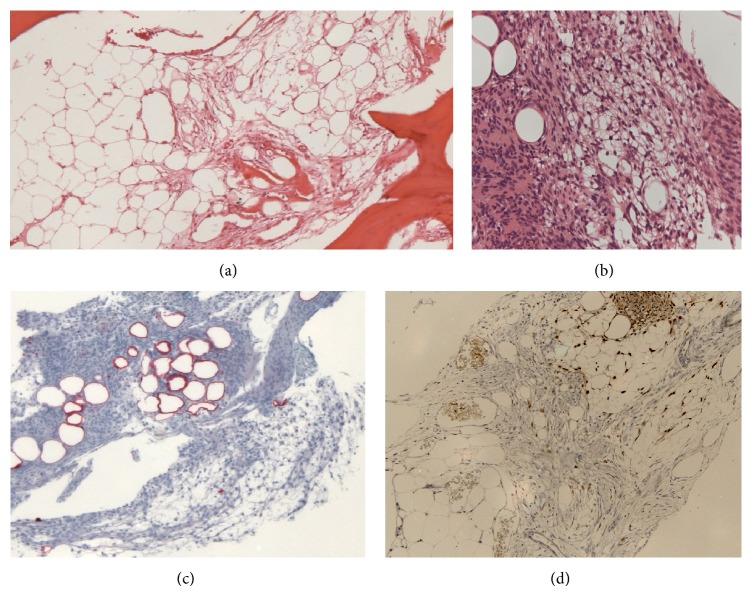
(a) Lipomatous meningioma with bone invasion and formation of cyst-like space (lower left). (b) Meningothelial foci interspersed with vacuolated lipomatous areas with cells resembling mature adipocytes. (c) Adipocyte-like cells stain for S100. (d) Interface between meningioma and normal fat shown by progesterone receptor staining (following decalcification process).

Histology of the skull flap revealed cyst-like spaces surrounded by meningioma with foci showing typical meningothelial morphology interspersed with areas composed of cells showing variable vacuolation (Figures 3(a) and 3(b)). Area of microcystic change was seen. Much of the lesion resembled adipose tissue. The adipocyte-like cells were labelled for S100 (Figure 3(c)) but also showed nuclear positivity for progesterone receptor similar to the meningothelial areas (Figure 3(d)). Interface between lipomatous tumour and marrow fat was readily demonstrated by progesterone receptor staining (Figure 3(d)). A small amount of tumour was attached to the dura with predominantly meningothelial features. The Ki-67 proliferative index was less than 0.5% (an experienced anatomical pathologist assessed this visually to determine the result). These features are consistent with a World Health Organization (WHO) grade I lipomatous meningioma.

## 2. Discussion

Lipomatous meningiomas are rare subtypes of meningiomas. First described by Bailey and Bucy in 1931 [1], “lipomatous” or “lipoblastic” refers to the cells observed in meningothelial neoplasms resembling adipocytes or lipoblasts, without the implication that those cells are immature or malignant [2]. The WHO classification of tumours categorizes lipomatous meningiomas to be a metaplastic form of meningioma [3], although there is ongoing debate regarding meningioma's pathogenesis as true metaplasia of the meningothelial cells or accumulation of lipids within the cells [4–6]. Lipomatous meningiomas are classically WHO grade I tumours with good prognosis following complete removal. There is very limited data on these tumours with less than 50 cases of lipomatous meningiomas being described throughout the literature [7], and to date none of them have been observed to have intraosseous extension.

Metaplastic lipomatous meningiomas can be defined as meningiomas with striking focal or widespread mesenchymal areas, including osseous, cartilaginous, lipomatous, myxoid, or xanthomatous tissue components [4]. Lipomatous meningiomas reveal low mitotic rates. In our case the Ki-67 was 0.5%, which is in keeping with the findings of Tang et al., 2013, in which 14 out of 15 metaplastic meningiomas showed Ki-67 rates of less than 1%, including the two lipomatous meningiomas [8]. Bone invasion can be seen in meningiomas, yet it is not considered as a part of the grading classification. Recently there have been suggestions that lipomatous meningiomas occur from lipid accumulation due to metabolic abnormality of the neoplastic cells rather than true metaplasia of the meningioma cells [9].

Throughout the English literature 41 cases of lipomatous meningiomas have been described [7], one associated with a secreting meningioma [10] and another with a cerebral arteriovenous malformation [11]. No cases were associated with intraosseous extension, although there has been a previous report of a meningioma occurring at the site of an intraosseous lipoma [12]. To our knowledge this represents the first reported case of a lipomatous meningioma with predominant intraosseous extension.
